# Medical Students Learning on the COVID-19 Front Line

**DOI:** 10.2196/28264

**Published:** 2021-06-01

**Authors:** Ioanna Zimianiti, Vyshnavi Thanaraaj, Francesca Watson, Oluwapelumi Osibona

**Affiliations:** 1 Imperial College London, School of Medicine London United Kingdom

**Keywords:** medical education, COVID-19, frontline workers, medical student, viewpoint, perspective, infectious disease, experience, barrier, motivation

## Abstract

In this viewpoint, we share our perspectives, as medical students at Imperial College London, on our experiences during our Infectious Diseases placement at Northwick Park Hospital, touching upon other students’ experiences at other sites as well. We highlight some of the main drivers of and barriers to medical students seeing patients with COVID-19.

Northwick Park Hospital, situated in North West London, was one of the most affected hospitals when the first COVID-19 lockdown began in the United Kingdom in March 2020 [1]. Its surrounding boroughs of Harrow and Brent have seen high infection rates from as early as March 2020. The site has both a tertiary infectious disease center and a large accident and emergency department and it was therefore designated as an additional high-consequence infectious disease intensive care unit (ICU) [2]. The end of 2020 and the beginning of 2021 were marked by a surge in COVID-19 admissions, which put an unprecedented strain on the health care system [3]. While much research regarding the disease and pandemic has been published, there is a lack of information about the impact of the pandemic on medical students’ learning and assistance on COVID-19 wards. This opinion piece reflects on our personal experiences during our Infectious Diseases placement at Northwick Park Hospital during this time and compares them to those of our peers at other sites.

During the first lockdown from March to July 2020, the COVID-19 pandemic was at one of its worst stages and the global picture was unclear. As Imperial College medical students, along with many others across the country, we were sent home and our clinical placements were suspended to reduce the risk of exposure to COVID-19 during this time. All classes were moved online, with lectures delivered over Zoom and Microsoft Teams [4]. However, from July 2020 onwards, more sustainable and effective plans for medical education were identified, which led to medical students being classified as key workers, allowing us to continue with clinical placements.

One of these placements is Infectious Diseases, which occurs as part of a 3-week block including aspects of genitourinary medicine and HIV. Conditions typically seen on these wards largely comprise tropical diseases such as typhoid and malaria. However, due to the increase in COVID-19 cases and subsequent travel restrictions, the incidence of such diseases, which are not endemic in Europe, has decreased [5].

Prior to our Infectious Diseases placement at Northwick Park Hospital, our exposure to patients with COVID-19 had been limited. However, with this placement coinciding with the second wave of the pandemic and with multiple suspected and confirmed cases on the Infectious Diseases ward, we were strongly encouraged to engage with such patients. This ranged from relaying necessary observations in the ward rounds to taking histories in order to help understand the various clinical manifestations of COVID-19, thus supporting our learning.

A key factor driving our motivation to engage with patients with COVID-19 was that our contact hours at previous placements had been reduced as part of social distancing measures; thus, we were more determined to maximize our learning experience. Furthermore, infection with COVID-19 can be an isolating experience for patients, as they are often not allowed visits from friends and family. As students, having more time than the busy medical team, we were able to spend more time with patients, which was greatly appreciated by patients and highly rewarding for us.

The national shortage of personal protective equipment (PPE) was something we were highly aware of due to comprehensive coverage by the news, especially at the start of the pandemic. On starting our placements, we were provided with gloves, surgical masks, and aprons (standard PPE) to see patients; nevertheless, our views were mixed on whether this was enough. This was largely due to experience in previous placements; some of us had previously been in ICUs where more significant PPE (eg, eyewear and scrub caps) was readily available, despite not being directly exposed to patients with COVID-19. However, some aerosol-generating procedures, including intubation and mechanical ventilation, were performed in the ICU area, which accounted for this heightened level of PPE. These worries were relieved for us through the team’s consensus that this was adequate protection, and clear posters on the wards that reinforced this message.

Speaking with our colleagues on Infectious Diseases placements at different hospitals in North West London, including St Mary’s, Hammersmith, Ealing, Charing Cross, and Chelsea and Westminster, we discovered their experiences and level of engagement with patients with COVID-19 varied. To understand this further, we disseminated a small survey, completed by 28 students, to assess their experiences.

Student experiences varied across the different sites, ranging from being encouraged to see patients with COVID-19 regularly, to being discouraged or choosing to opt out due to concerns about putting themselves or their loved ones at risk. Some students decided against contact because they felt it was an unnecessary risk as they were not contributing to patient care directly, and the experience was not aiding their learning. On the other hand, we found this to be an insightful learning experience, as we were greatly encouraged to play an active role during ward rounds. In addition, the Infectious Diseases team at Northwick Park Hospital generally encouraged engagement and followed up our encounters on the ward with case-based discussions, multidisciplinary team meetings, and X-ray meetings.

The students that were exposed to patients with COVID-19 largely felt they had adequate PPE, stating that “consultants assured us it was okay,” “the whole team was wearing the same PPE,” and emphasizing that maintaining physical distance and practicing good hand hygiene contributed to their comfort. However, other students felt the standard PPE was inadequate as, similar to our experience, they had received more significant PPE while on prior placements. This highlights that there is room for further, detailed communication and discussions with students regarding the levels of PPE required in different areas of the hospital, as this may encourage participation.

Students felt their comfort levels improved with exposure to patients over the course of the placement, but they felt it did not have a significant impact on their fifth year learning experience. The former is something we can relate to ourselves, as we felt that seeing patients with COVID-19 reduced our fear of being around these patients during the pandemic, as long as we were wearing adequate PPE.

In terms of education, the COVID-19 pandemic resulted in many clinical and nonclinical tutors being redirected to their health care roles and there was a subsequent reduction of teaching on the wards. Since this placement was our first contact with patients with COVID-19, the support of the medical team was crucial in guiding and encouraging our decisions to engage in the management of patients with COVID-19, as well as in alleviating any underlying worries. As we have become more involved in the “frontline” experience, we have found we can still get the necessary clinical exposure, alleviating uncertainty and equipping us with the required skills to manage similar situations when we qualify as doctors. Overall, we can all agree that our experiences made this placement—which at first appeared daunting and concerning in light of the current pandemic—an extremely interesting and enjoyable one.

## Abbreviations

ICU: intensive care unit
PPE: personal protective equipment
